# L-Proline Supplementation Alleviates Lipopolysaccharide-Induced Intestinal Inflammation in Weaned Rabbits by Modulating Mucosal Barrier Function and Gut Microbiota

**DOI:** 10.3390/biology15171482

**Published:** 2026-09-01

**Authors:** Biyan He, Siming Tao, Yiling Xiang, Caixue Xu, Yongtian Yin, Xiaoxian He, Xiaokang Ma, Zhenlong Wu, Yinghe Qin, Ning Liu

**Affiliations:** 1 State Key Laboratory of Animal Nutrition and Feeding, China Agricultural University, Beijing 100193, China; biyanho@cau.edu.cn (B.H.); 18045159167@163.com (S.T.); zxy13098561165@email.swu.edu.cn (Y.X.); 20220200217@stu.qau.edu.cn (C.X.); a05220200@neau.edu.cn (Y.Y.); sy20253041161@cau.edu.cn (X.H.); wuzhenlong@cau.edu.cn (Z.W.); qinyinghe@cau.edu.cn (Y.Q.); 2 College of Animal Science and Technology, Hunan Agricultural University, Changsha 410128, China; maxiaokang@hunau.edu.cn

**Keywords:** L-proline, weaned rabbits, intestinal mucosal barrier, gut microbiota, intestinal inflammation

## Abstract

**Simple Summary:**

Weaning is a critical and stressful period in commercial rabbit production. During this period, young rabbits are highly susceptible to intestinal inflammation and diarrhea, which can lead to growth retardation, increased mortality, and substantial economic losses. Lipopolysaccharide (LPS), a component derived from pathogenic Gram-negative bacteria in the gut, is widely used to induce intestinal inflammatory injury. This study demonstrated that supplementation with 1% L-proline through drinking water alleviated intestinal tissue damage, reduced inflammatory responses, and was associated with remodeling gut microbial communities in LPS-challenged weaned rabbits. These findings offer a practical nutritional strategy for improving intestinal health and alleviating weaning-associated stress in commercial rabbit production.

**Abstract:**

L-proline (Pro) is a conditionally essential amino acid that has been reported to exert protective effects on intestinal health. This research investigated whether Pro supplementation reduces intestinal inflammation in weaned rabbits, and whether this effect involves the maintenance of the intestinal mucosal barrier and the composition of gut microbiota. A total of thirty weaned New Zealand White rabbits were randomly divided into five groups: a control group, an LPS-challenged model group, and three LPS-challenged groups receiving 0.5%, 1% or 2% Pro in their drinking water. Following the overall results of the study, 1% Pro was chosen for further investigation. Notably, 1% Pro supplementation significantly decreased the spleen index, alleviated colonic histopathological injury, enhanced the expression of the tight junction proteins Occludin and Zonula Occludens-1 (ZO-1), restored the population of goblet cells, decreased the colonic mRNA levels of the pro-inflammatory cytokines *IL1B* and *IL6*, and increased the expression of the anti-inflammatory cytokine *IL10*. Microbiome analysis revealed that Pro supplementation was associated with alterations in the dysbiotic gut ecosystem, including increased relative abundances of potentially beneficial genera such as *Ruminococcus, Christensenellaceae_R-7_group*, and *Lachnospiraceae_NK4B4_group*, and decreased the relative abundance of opportunistic pathogens like *Escherichia–Shigella*. Overall, these results suggest that Pro functions as a microbiota-modulating immunonutrient that mitigates intestinal inflammation and supports the integrity of the mucosal barrier, likely through changes in gut microbial composition.

## 1. Introduction

Rabbit production constitutes an indispensable component of sustainable small-scale livestock farming, as rabbit meat is valued for its high-quality protein content, low fat content, and low cholesterol level. However, rabbit production is constrained by a persistent physiological bottleneck associated with the weaning transition. In rabbits, weaning involves an abrupt shift from highly digestible maternal milk to a complex solid diet, accompanied by environmental stress and the loss of maternally derived passive immunity [1,2]. As a result, this stage is commonly linked to post-weaning intestinal inflammation, a multifactorial condition that can cause severe diarrhea, growth retardation, and high mortality rates, often exceeding 30–50% in intensive farming systems [3]. The gastrointestinal tract and immune system of weaned rabbits are functionally immature, rendering them particularly susceptible to Gram-negative bacterial endotoxins [4].

Lipopolysaccharide (LPS) is a key structural element found in the outer membrane of Gram-negative bacteria and a strong trigger for acute inflammation in the intestines. It compromises the integrity of the mucosal barrier, activates excessive inflammatory responses, and disturbs gut microbial homeostasis, thereby contributing to diarrhea, growth retardation, and increased mortality [5,6,7]. As a typical pathogen-associated molecular pattern (PAMP), LPS is recognized by Toll-like receptor 4 (TLR4) expressed on intestinal epithelial cells and macrophages [8,9]. This recognition activates canonical intracellular inflammatory pathways, particularly the nuclear factor kappa-light-chain-enhancer of activated B cells (NF-κB) and mitogen-activated protein kinase (MAPK) signaling pathways, thereby triggering a deleterious signaling cascade, promoting the excessive production of pro-inflammatory mediators, including tumor necrosis factor-α (TNF-α), interleukin-6 (IL-6), and interleukin-1β (IL-1β) [10,11], ultimately leading to epithelial barrier disruption. Dysbiosis of the gut microbiota could exacerbate this inflammatory pathway by increasing the release of endogenous LPS and facilitating its translocation across the intestinal epithelium [12]. In studies with rabbits, exposure to LPS markedly downregulated the expression of tight junction proteins such as Occludin, Claudin-1, and Zonula Occludens-1 (ZO-1), which in turn heightened paracellular permeability [13]. The translocation of luminal toxins into the bloodstream further intensifies oxidative stress and tissue injury. Consequently, the LPS-challenged rabbit model is valuable for assessing the protective effects of functional nutrients against intestinal damage. Given the considerable economic impact of intestinal inflammation in weaned rabbits, it is essential to develop targeted nutritional strategies aimed at reinforcing the intestinal barrier. Therefore, nutritional approaches that bolster intestinal resilience could be a vital strategy for promoting sustainable rabbit farming.

L-proline (Pro) has long been considered a nonessential amino acid for monogastric animals [14]. Nevertheless, it is now increasingly viewed as a conditionally essential amino acid, particularly during periods of rapid growth and immune challenges. As a distinctive amino acid, Pro plays several vital roles in intestinal physiology. It acts as a key precursor in collagen synthesis [15], which is crucial for maintaining the structural integrity of the intestinal basement membrane, and it can influence cellular redox status through the proline oxidase (POX) pathway [16,17]. Accumulating evidence has demonstrated the critical role of Pro in intestinal homeostasis. Previous investigations have shown that exogenous Pro supports the metabolic fitness of group 3 innate lymphoid cells and promotes their secretion of interleukin-22, a cytokine that facilitates epithelial repair and antimicrobial defense [18]. In studies involving rabbits, Pro has been shown to offer protective benefits against heat stress and oxidative damage, suggesting its roles extend beyond protein deposition to include the regulation of stress responses and immune regulation [19].

The rabbit gastrointestinal tract contains a highly developed cecum that harbors a dense and diverse microbial community essential for fiber fermentation and volatile fatty acid production. The gut microbiota serves as a critical biological barrier, but it is highly vulnerable to disruption during the weaning transition. Dysbiosis linked to weaning in rabbits is often marked by a decline in beneficial bacteria, like *Lactobacillus* and *Bacteroides*, alongside an increase in opportunistic pathogens, including members of *Proteobacteria* and *Escherichia coli* [20,21]. Growing research suggests that there are reciprocal relationships between amino acid metabolism and the gut microbiota in swine and murine models [16,22]. However, these results may not be applicable to rabbits because of their unique cecal fermentation physiology. Specifically, the role of Pro in alleviating LPS-induced intestinal inflammation in weaned rabbits, particularly through mechanisms related to the microbiome, is not yet fully understood.

Therefore, this research aimed to investigate whether Pro supplementation through drinking water can reduce LPS-induced intestinal inflammation in weaned rabbits. We assessed the impact of Pro on various factors, including systemic inflammatory status, colonic morphology, mucosal barrier integrity, inflammatory cytokine mRNA expression, and gut microbial composition, using high-throughput 16S rRNA gene sequencing. These findings provide evidence supporting the potential application of Pro as a functional nutritional additive to enhance intestinal health in weaned rabbits.

## 2. Materials and Methods

### 2.1. Experimental Materials

New Zealand White rabbits were sourced from SPF (Beijing) Biotechnology Co. Ltd. (Beijing, China). LPS and Pro were purchased from Sigma (St. Louis, MO, USA).

### 2.2. Experimental Methods

Male weaned New Zealand White rabbits were housed in the rabbit facility of China Agricultural University under a daily 12-h light/12-h dark cycle. The ambient temperature was maintained at 22–25 °C, and the relative humidity was controlled at 50–60%. Feeding occurred twice daily at 8:00 a.m. and 5:00 p.m., and the rabbits had ad libitum access to water. We freshly prepared the Pro-containing drinking water every morning and replaced it daily at the same time to maintain stability and prevent bacterial contamination. The basal diet was purchased from Xingtai Xiangyang Feed Co., Ltd. (Xingtai, China), with the nutritional details outlined in Table 1 from the feed label. The detailed composition of the raw materials used in this study is listed in Table S2. The body weight of the rabbits was recorded throughout the duration of the experiment.

### 2.3. Experimental Design

#### 2.3.1. Animal Experiments

**Figure 1:**
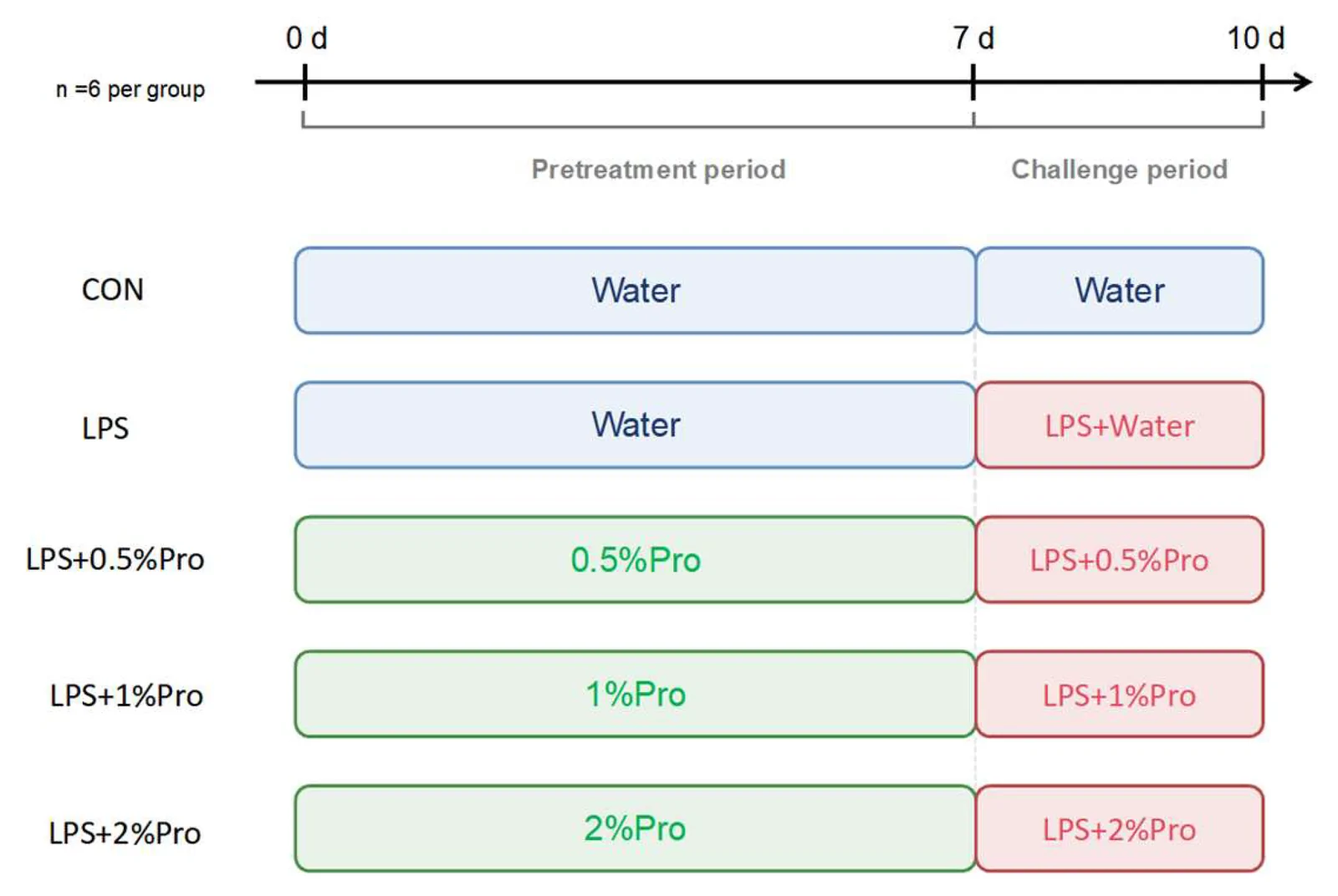
Schematic diagram of the experimental design.

Thirty 35-day-old male weaned New Zealand White rabbits (1.0–1.2 kg) were randomly assigned to five experimental groups (*n* = 6 per group): Control, LPS, and three Pro treatment groups receiving 0.5%, 1%, or 2% Pro in drinking water. All animal procedures were approved by the Welfare and Ethical Committee for Experimental Animal Care of China Agricultural University (AW20206202-1-16). The experiment lasted 10 days. From days 8 to 10, rabbits in the LPS and LPS + Pro groups were administered daily intraperitoneal injections of LPS (1.5 mg/kg) to induce intestinal inflammation. Meanwhile, Pro was provided in the drinking water at the specified concentrations (0.5%, 1%, or 2%) throughout the 10-day experiment (both during the pretreatment period and the LPS-challenging period). Rabbits in the Control group received an equivalent volume of sterile physiological saline by intraperitoneal injection on days 8 to 10. The experimental design is illustrated in Figure 1.

#### 2.3.2. Sample Collection

At the end of the trial, blood samples were collected and the rabbits were euthanized by exsanguination under anesthesia in accordance with the approved institutional animal care protocol. The entire colon was excised and its length was measured. Colonic tissues and luminal contents were immediately separated, frozen in liquid nitrogen, and stored at −80 °C. Blood samples were centrifuged at 1200× *g* for 20 min, and the resulting serum samples were stored at −80 °C.

#### 2.3.3. Histopathological Assessment

Colonic segments were fixed in 4% paraformaldehyde for 24 h, dehydrated, and embedded in paraffin. Paraffin sections (5 μm thick) were prepared using a Thermo Fisher Scientific microtome (San Jose, CA, USA) and stained with hematoxylin and eosin (H&E) to evaluate tissue architecture and mucosal injury. Microscopic lesions were assessed by an independent evaluator blinded to group allocation using established scoring criteria [23]. The intestinal inflammation score, with a maximum score of 12, was calculated as the sum of three parameters: (1) loss of surface epithelial cells, (2) crypt destruction, and (3) inflammatory cell infiltration. Each parameter was scored from 1 to 4. The scoring scheme is presented in Table 2. Goblet cells were visualized by using an Alcian blue–periodic acid–Schiff (AB-PAS) Stain Kit (Solarbio, Beijing, China).

#### 2.3.4. Immunofluorescence

Paraffin-embedded colonic sections were deparaffinized in xylene, rehydrated through a graded ethanol series, and subjected to antigen retrieval using citrate antigen retrieval solution in a heated water bath for 10 min. After cooling and washing with phosphate-buffered saline (PBS), sections were blocked with 5% bovine serum albumin for 60 min to reduce nonspecific binding. Sections were then incubated with anti-ZO-1 antibody (21773-1-AP, Proteintech; 1:100) overnight at 4 °C, followed by washing with PBST (PBS containing 0.1% Triton X-100). After incubation for 1 h with Alexa Fluor 555-labeled secondary antibody (A21428, Thermo Fisher Scientific; 1:200), the sections were washed in the dark and mounted with antifade medium containing DAPI (Beyotime, Beijing, China). The negative control for this staining is presented in Figure S2. Fluorescence images were acquired using a Carl Zeiss Axio Scope A1 microscope (Zeiss, Göttingen, Germany) equipped with a 20× objective. For each section, 5 randomly selected, non-overlapping fields from intact colonic mucosa were analyzed using ImageJ 1.54g (National Institutes of Health, Bethesda, MD, USA). Fields containing tissue folds, section edges, or saturated signals were excluded. The ZO-1 fluorescence was quantified as mean fluorescence intensity.

#### 2.3.5. Western Blot

Immunoblotting was performed according to standard protocols. In summary, colonic samples were lysed using RIPA buffer (Beyotime, Beijing, China) supplemented with 1% protease and phosphatase inhibitor cocktails. Protein concentrations were determined using a BCA Protein Assay Kit (Beyotime). Equal amounts of protein were separated by SDS-PAGE and transferred onto polyvinylidene fluoride (PVDF) membranes. After blocking with 5% nonfat milk for 1 h at room temperature, membranes were incubated overnight at 4 °C with primary antibodies. Protein bands were visualized using enhanced chemiluminescence reagents and quantified by densitometric analysis with ImageJ software, with β-actin used as the loading control. The primary antibodies used were rabbit anti-Occludin (Proteintech, 27260-1-AP; 1:2000) and mouse anti-β-actin (Abcam, ab49900; 1:20,000). The original, uncropped western blot images are available in Figure S1.

#### 2.3.6. qPCR Assay

Total RNA extraction from colonic tissue was carried out utilizing TRIzol reagent (Thermo Fisher Scientific). cDNA was synthesized using TranScript All-in-One First-Strand cDNA Synthesis SuperMix. Quantitative PCR was performed on a 7900HT Fast Real-Time PCR System (Applied Biosystems, San Francisco, CA, USA) using TransStart Tip Green qPCR SuperMix (TransGene Biotech, Beijing, China). Each 20 µL reaction system contained 10 µL of 2× SuperReal PreMix Plus, 0.6 µL of forward primer, 0.6 µL of reverse primer, 2 µL of cDNA template, and 6.8 µL of nuclease-free water. The thermal cycling conditions were as follows: initial denaturation at 95 °C for 15 min, followed by 40 cycles of denaturation at 95 °C for 10 s, annealing at 60 °C for 20 s, and extension at 72 °C for 32 s. After amplification, a melting curve analysis was conducted to verify the specificity of the products. Each sample underwent three technical replicates. The relative mRNA expression levels of *IL1B*, *IL6*, and *IL10* were normalized to the reference gene GAPDH and determined using the 2^−ΔΔCt^ method. The primer sequences can be found in Table 3.

#### 2.3.7. Colonic Microbiota Analysis

Total microbial genomic DNA was extracted from colonic contents using the E.Z.N.A.^®^ soil DNA Kit (Omega Bio-tek, Norcross, GA, USA) according to the manufacturer’s instructions. The V3-V4 hypervariable region of the bacterial 16S rRNA gene was amplified using primer pairs 338F (5′-ACTCCTACGGGAGGCAGCAG-3′) and 806R (5′-GGACTACHVGGGTWTCTAAT-3′) by T100 Thermal Cycler PCR thermocycler (BIO-RAD, Hercules, CA, USA). Purified amplicons were pooled in equimolar amounts and paired-end sequenced on an Illumina Nextseq2000 platform (Illumina, San Diego, CA, USA) according to the standard protocols by Majorbio Bio-Pharm Technology Co. Ltd. (Shanghai, China). After demultiplexing, sequences were quality-filtered using fastp [24] (version 0.23.4) and merged using FLASH [25] (version 1.2.11). High-quality sequences were denoised using DADA2 plugin in the Qiime2 (version 2020.2) with recommended parameters to obtain amplicon sequence variants (ASVs) [26,27]. Subsequent bioinformatic analyses were performed based on the ASV dataset. The detailed sequencing statistics and ASV counts for each sample are provided in Table S1.

### 2.4. Statistical Analysis

Statistical analysis was performed using SPSS 22.0 (IBM, Armonk, NY, USA). Prior to one-way ANOVA, the normality of residuals was assessed using the Shapiro–Wilk’s test, and the homogeneity of variances was verified using Levene’s test. For growth performance, spleen indices, colon length, histological scores, Western blotting, immunofluorescence intensity, and qPCR data, differences among groups were analyzed by one-way ANOVA followed by Tukey’s multiple comparison test. A *p*-value of <0.05 was considered statistically significant. For beta-diversity analysis, principal coordinate analysis (PCoA) was performed based on Bray–Curtis distances, with group differences evaluated using both Analysis of Similarity (ANOSIM) and PERMANOVA (Adonis). Differentially enriched bacteria taxa were based on the Kruskal–Wallis test. Spearman’s rank correlation coefficient was used to assess relationships between bacterial genera and cytokine levels.

## 3. Results

### 3.1. Proline Ameliorates LPS-Induced Intestinal Inflammation

**Figure 2:**
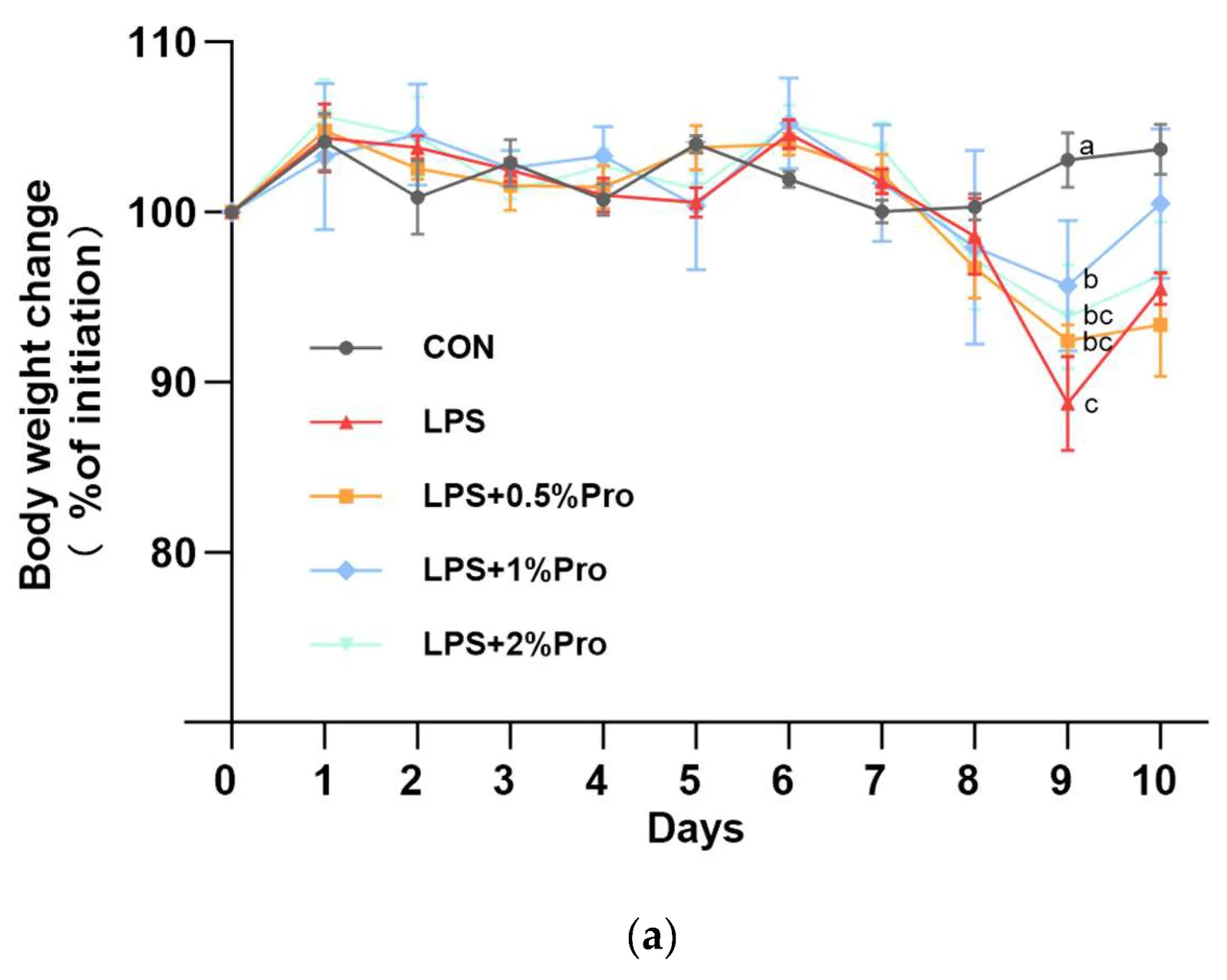

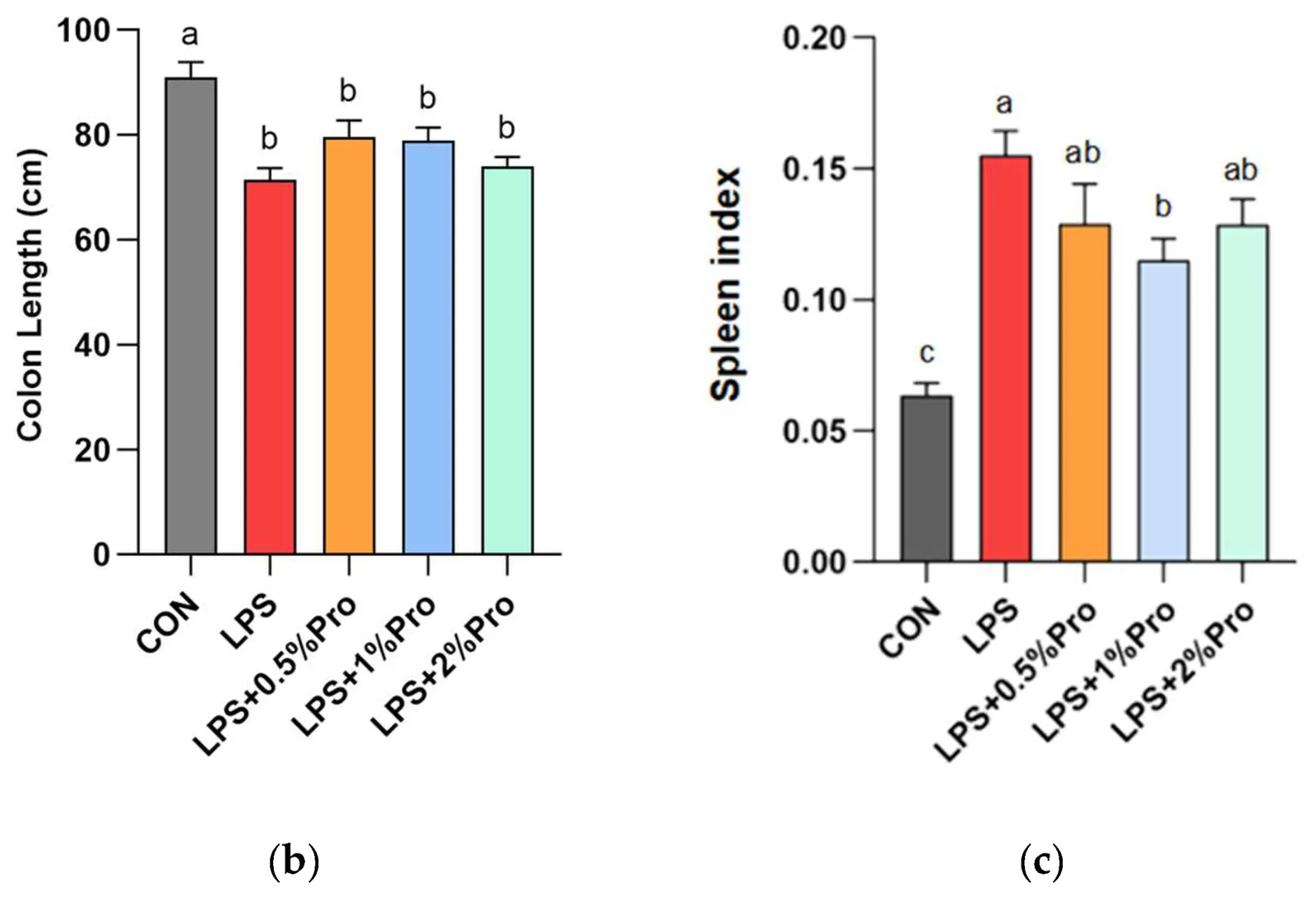
Pro supplementation ameliorates clinical symptoms. (**a**) Changes in body weight presented as a percentage of the initial weight (*n* = 6). (**b**) Quantification of colon length (*n* = 6). (**c**) Spleen index, an indicator of systemic immune activation (*n* = 6). Data are presented as means ± standard error of the mean (SEM). Different letters above the bars indicate significant differences among groups (*p* < 0.05). Groups sharing the same letter are not significantly different from each other.

To evaluate the therapeutic effects of Pro, a rabbit model of intestinal inflammation induced by LPS was created. In comparison to the Control group, LPS challenge resulted in an approximately 10% decrease in body weight among weaned rabbits (*p* < 0.05), reflecting a significant systemic effect of acute inflammatory stress. Rabbits receiving 1% Pro supplementation experienced notably less body weight loss than those in the LPS group (*p* < 0.05), indicating that Pro mitigated some of the detrimental impacts of LPS on growth performance (Figure 2a). In contrast, supplementation with 0.5% and 2% Pro had no significant effect on body weight compared with the LPS group. Alongside the weight changes, the LPS challenge also led to clear macroscopic pathological changes. The LPS-treated rabbits exhibited colonic shortening (*p* < 0.05), a common marker of intestinal inflammation severity (Figure 2b). However, none of the Pro treatments significantly improved colon length. The spleen index showed a marked increase following LPS treatment (*p* < 0.05), indicating a heightened systemic immune and inflammatory responses, while the 1% Pro treatment significantly lowered the spleen index compared to the LPS group (Figure 2c). The 0.5% and 2% Pro treatment groups had no significant effect on the spleen index compared with the LPS group. Given the overall impact on the assessed parameters, 1% Pro was chosen for further investigation. In summary, these results suggest that Pro supplementation partially mitigated the negative effects of LPS exposure in weaned rabbits.

### 3.2. Proline Preserves Colonic Morphology and Barrier Integrity in LPS-Challenged Rabbits

**Figure 3:**
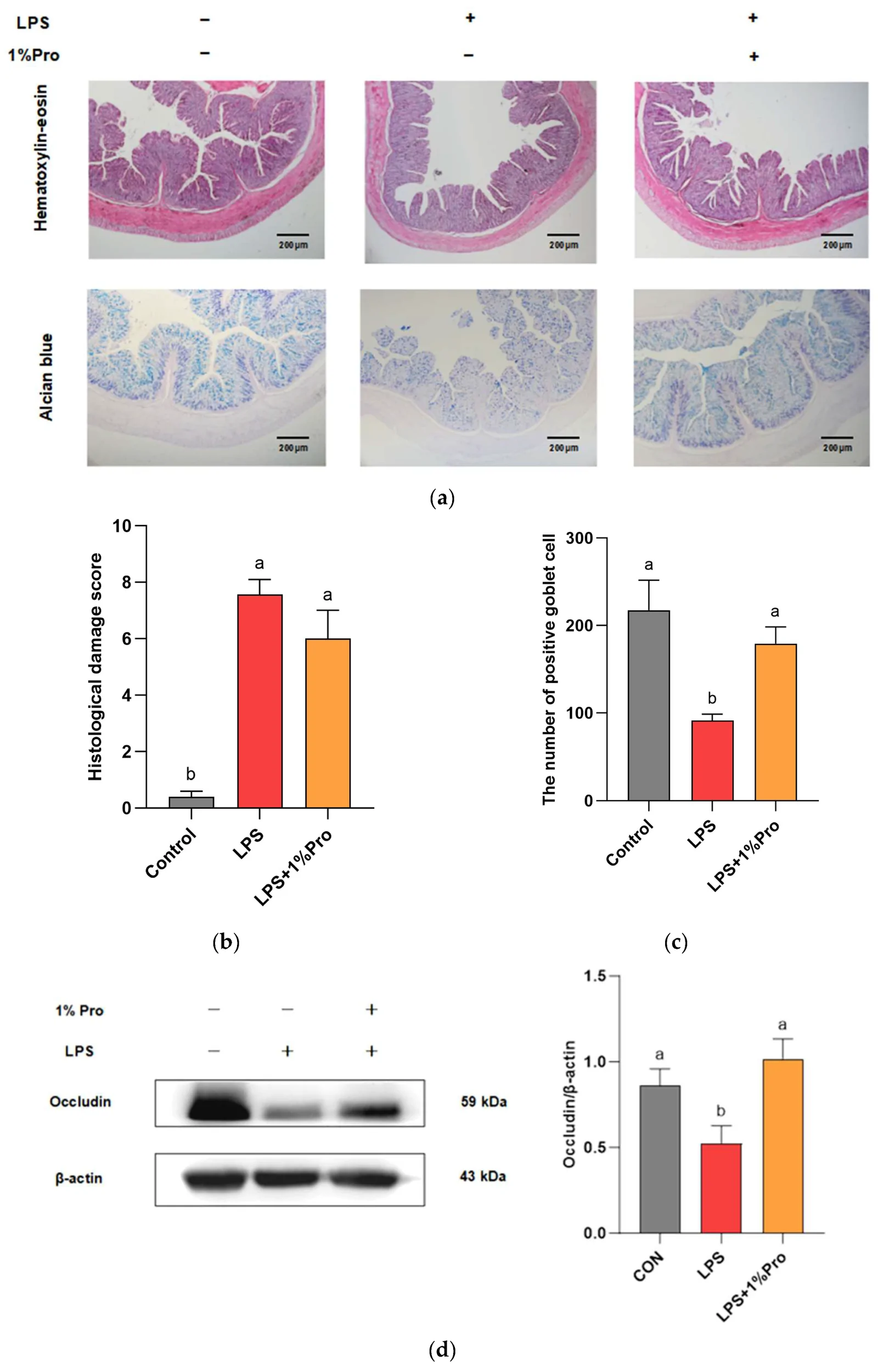

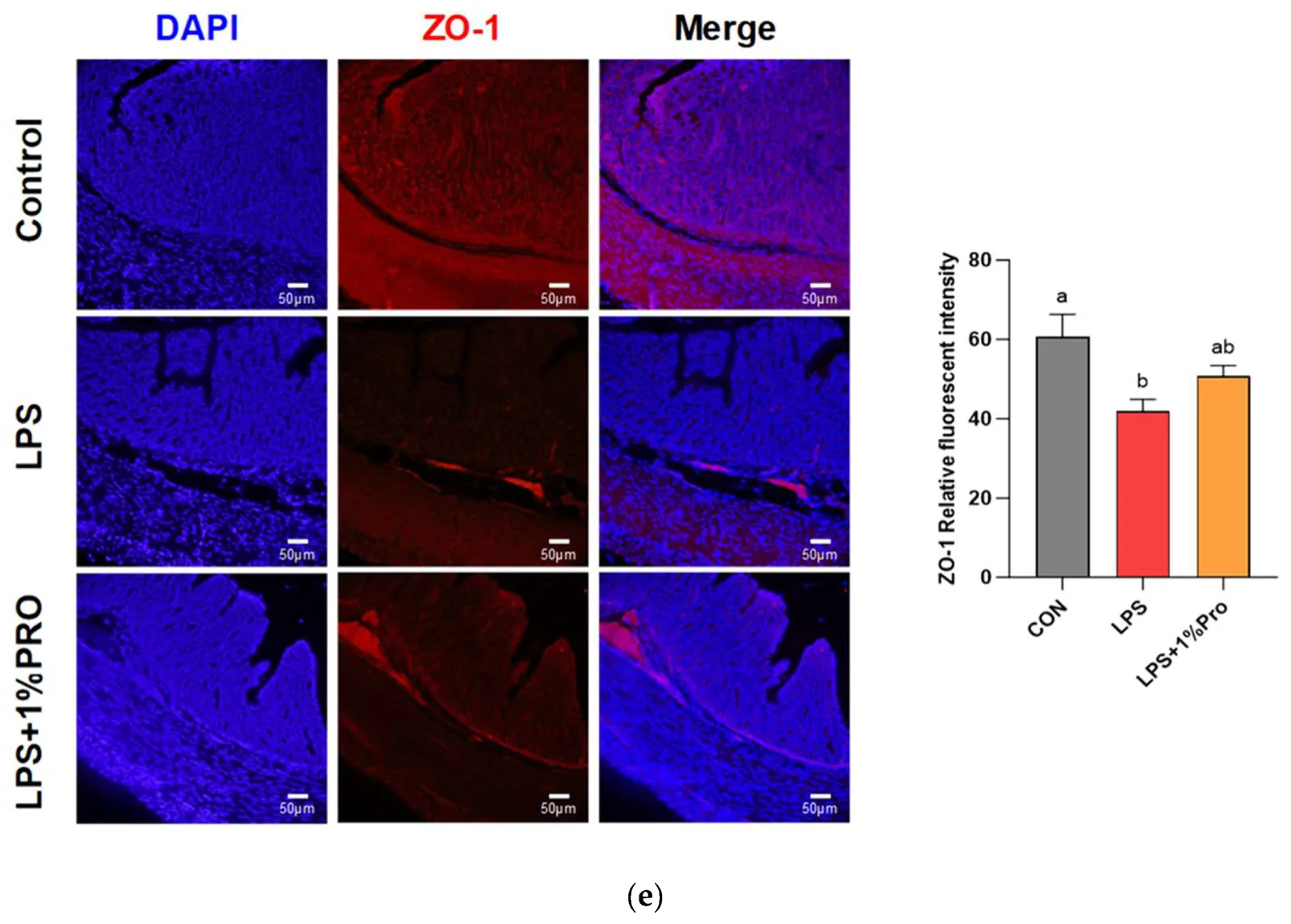
Pro supplementation alleviates pathological damage in LPS-induced intestinal inflammation. (**a**) Representative H&E- and AB-PAS-stained colonic sections showing mucosal architecture and inflammatory cell infiltration (scale bar: 200 μm). In the row labels, the minus sign (−) indicates no treatment and the plus sign (+) indicates the treatment with LPS or Pro. (**b**) Quantitative analysis of the histological findings (*n* = 6). (**c**) Goblet cell counts, calculated as the absolute number of goblet cells per unit area (*n* = 6). (**d**) Relative expression of Occludin normalized to β-actin (*n* = 6). (**e**) Representative immunofluorescence images and quantitative analysis of ZO-1 expression in colonic sections (*n* = 6, scale bar: 50 μm). In the row labels, the minus sign (−) indicates no treatment and the plus sign (+) indicates the treatment with LPS or Pro. Data are presented as means ± SEM. Different letters above the bars indicate significant differences among groups (*p* < 0.05). Groups sharing the same letter are not significantly different from each other.

Histological analysis of colon samples revealed that the Control group maintained a normal mucosal structure, with well-formed crypts, and minimal infiltration of inflammatory cells (Figure 3a). Conversely, the LPS treatment led to a breakdown of epithelial integrity and crypt architecture, along with significant inflammatory cell presence in both mucosa and submucosa. Correspondingly, the histological inflammation score was notably higher in the LPS group compared to the Control group (*p* < 0.05). Rabbits treated with 1% Pro demonstrated enhanced mucosal structure, decreased inflammatory cell presence, and a lower histological inflammation score relative to the LPS group (Figure 3b). Alcian blue staining indicated a significant reduction in goblet cell numbers due to LPS treatment (*p* < 0.05), while Pro supplementation effectively restored the goblet cell count (*p* < 0.05), suggesting an improvement in mucus barrier function (Figure 3c). To evaluate intestinal barrier integrity further, the expression levels of tight junction proteins were analyzed (Figure 3d). LPS treatment resulted in decreased Occludin expression (*p* < 0.05), whereas the addition of 1% Pro significantly increased Occludin levels compared to the LPS group (*p* < 0.05). Supporting the Western blot results, ZO-1 immunofluorescence staining revealed diminished junctional fluorescence in the LPS group compared to the Control group (*p* < 0.05). Pro supplementation restored the intensity of ZO-1 fluorescence slightly (Figure 3e). Overall, these findings indicate that Pro supplementation attenuated LPS-induced damage in the colon and was linked to enhanced markers of epithelial barrier function.

### 3.3. Proline Attenuates Intestinal Inflammation by Regulating Inflammatory Cytokine Expression

**Figure 4:**
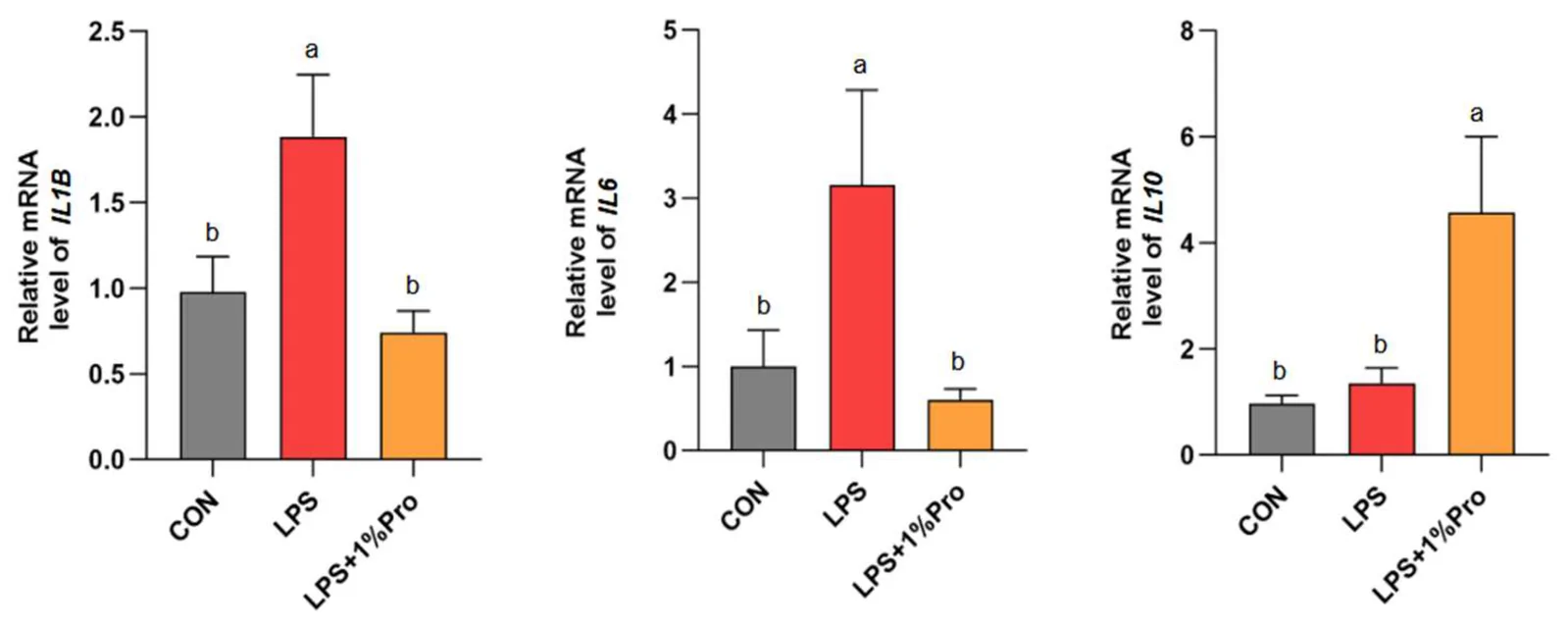
Relative mRNA expression of pro-inflammatory and anti-inflammatory cytokines (*n* = 6). Data are presented as means ± SEM. Different letters above the bars indicate significant differences among groups (*p* < 0.05). Groups sharing the same letter are not significantly different from each other.

To determine if the protective role of Pro was linked to changes in the intestinal inflammatory response, the mRNA expression levels of representative cytokines were measured in colonic tissues (Figure 4). As anticipated, LPS challenge triggered a significant inflammatory response, marked by heightened levels of pro-inflammatory cytokines and a disturbance in local immune homeostasis. Specifically, in comparison to the Control group, LPS significantly increased the mRNA expression levels of *IL1B* and *IL6* by 1.88-fold and 3.15-fold (*p* < 0.05), respectively. The addition of 1% Pro significantly reduced the mRNA levels of *IL1B* and *IL6* when compared to the LPS group (*p* < 0.05). Furthermore, Pro supplementation resulted in an increase in *IL10* levels compared to the LPS challenge alone (*p* < 0.05). The rise in *IL10* suggests that Pro may bolster anti-inflammatory effects and help restore immune balance in the intestine. Overall, these findings suggest that Pro supplementation is linked to a more favorable profile of colonic inflammatory cytokines in rabbits subjected to LPS challenges.

### 3.4. Proline Reshapes Gut Microbial Community Structure

**Figure 5:**
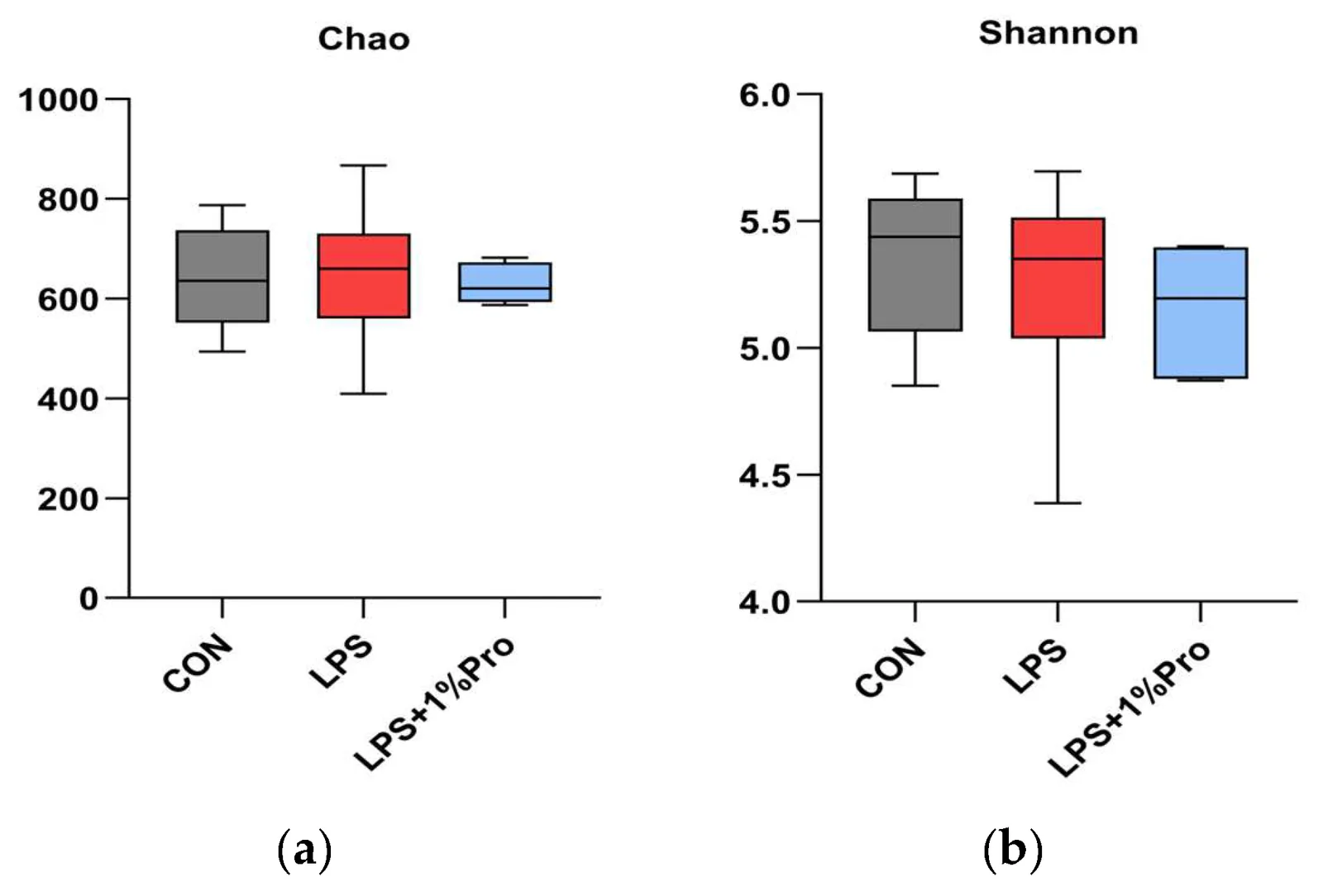

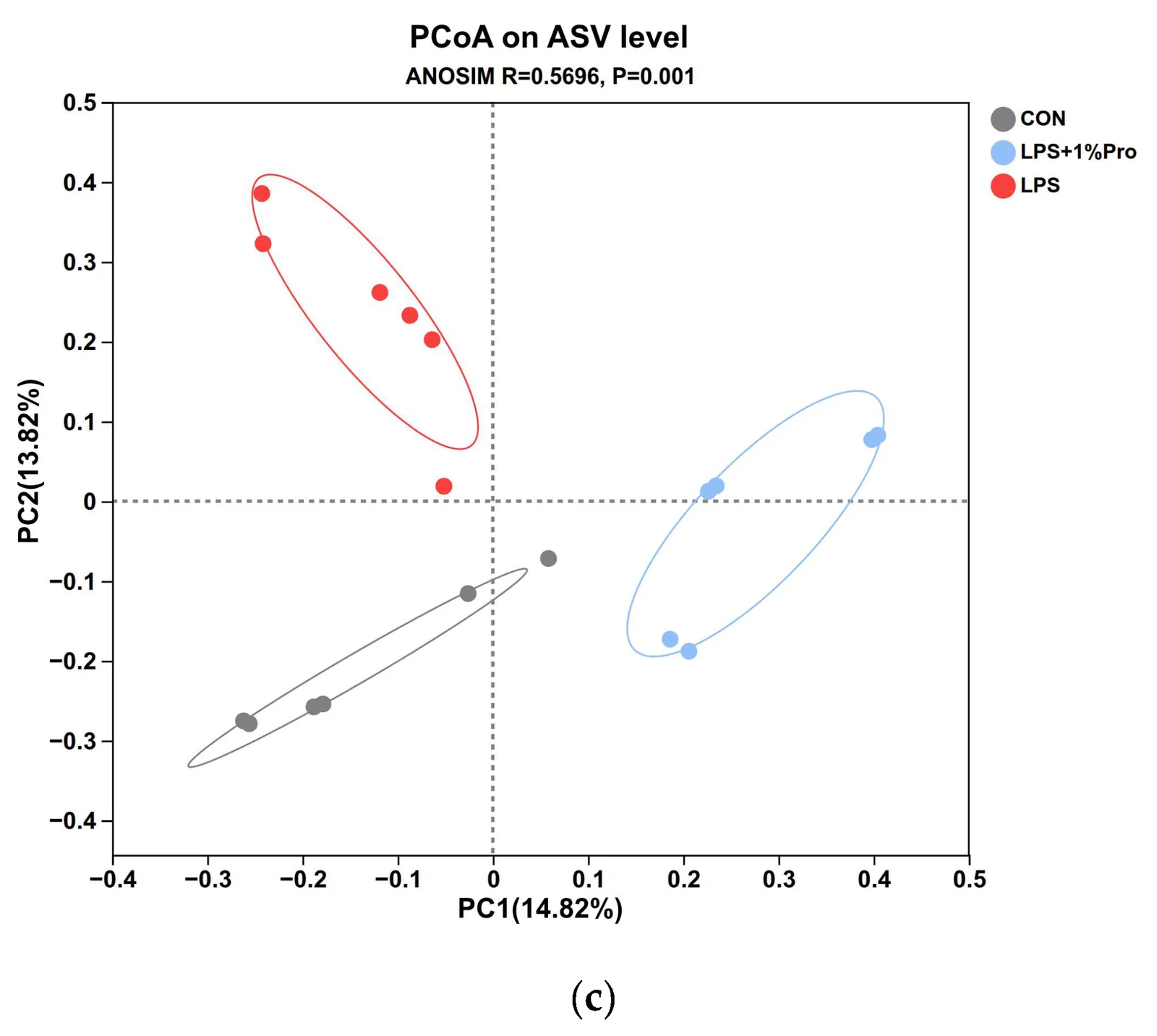
Pro treatment alters colonic microbial diversity and community structure in LPS-challenged rabbits. (**a**,**b**) Alpha-diversity analysis based on the Chao1 richness and Shannon evenness indices. Data are presented as means ± SD. (**c**) PCoA based on Bray–Curtis distances, showing structural separation among groups (ANOSIM R = 0.5696, *p* = 0.001; PERMANOVA R^2^ = 0.2577, *p* = 0.001).

To assess if the therapeutic benefits of Pro were linked to changes in gut microbiota, 16S rRNA gene sequencing was conducted on the contents of the colon. Alpha-diversity analysis revealed no notable differences in the Chao1 and Shannon indices among the groups tested (Figure 5a,b), suggesting that neither the LPS challenge nor Pro treatment significantly impacted the overall microbial richness or diversity. PCoA combined with ANOSIM (R = 0.5696, *p* = 0.001) and PERMANOVA (R^2^ = 0.2577, *p* = 0.001), demonstrated a distinct separation between the Control and LPS groups, indicating that the LPS challenge profoundly altered overall microbial composition (Figure 5c). In contrast, the microbiota of rabbits treated with Pro exhibited a notable shift in ordination space, clustering away from the LPS group and aligning more closely with the Control group. These results imply that Pro supplementation was linked to alterations in the gut microbial community structure in rabbits subjected to LPS challenges.

### 3.5. Proline Restores Gut Microbial Composition and Enriches Beneficial Bacteria

**Figure 6:**
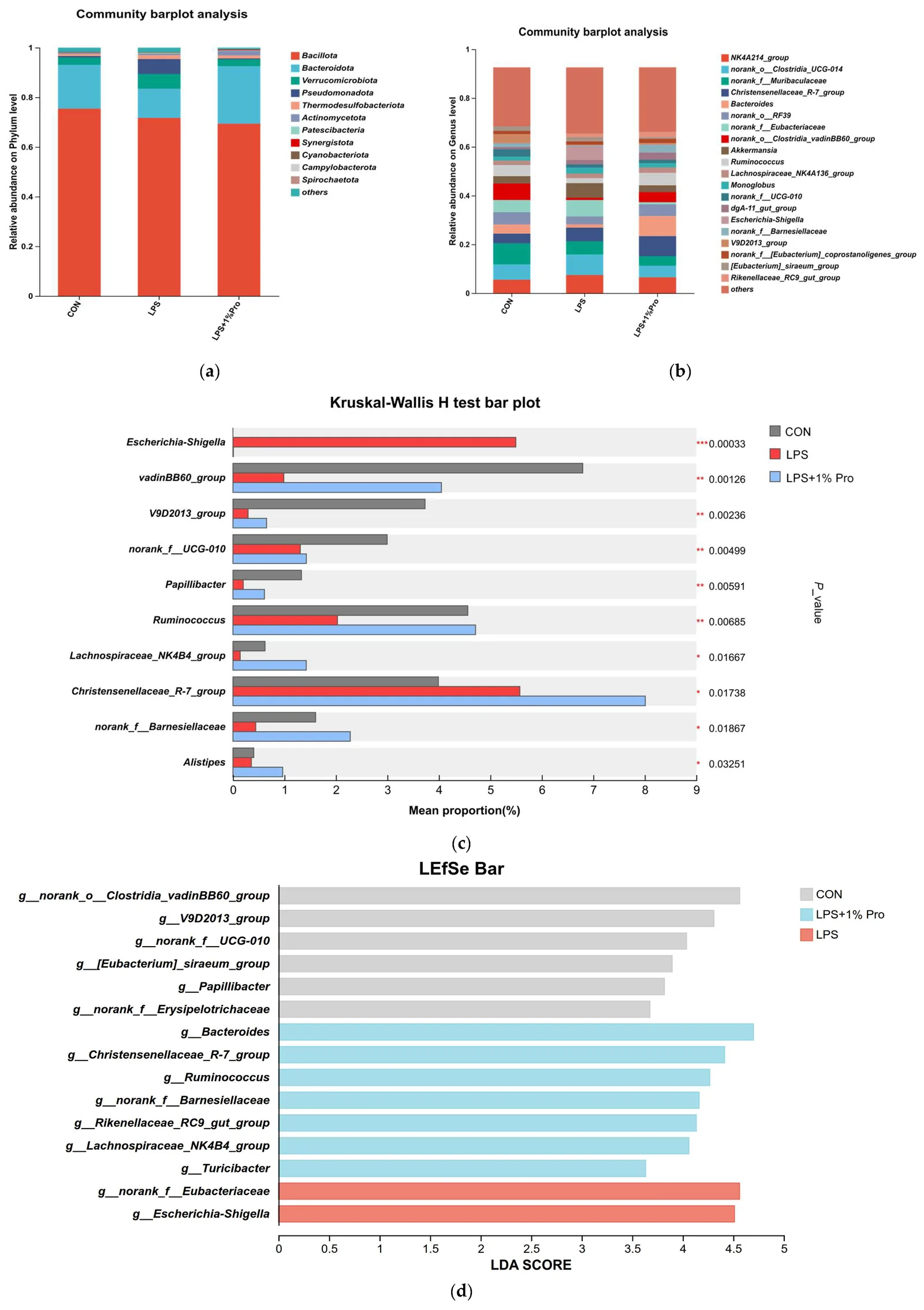

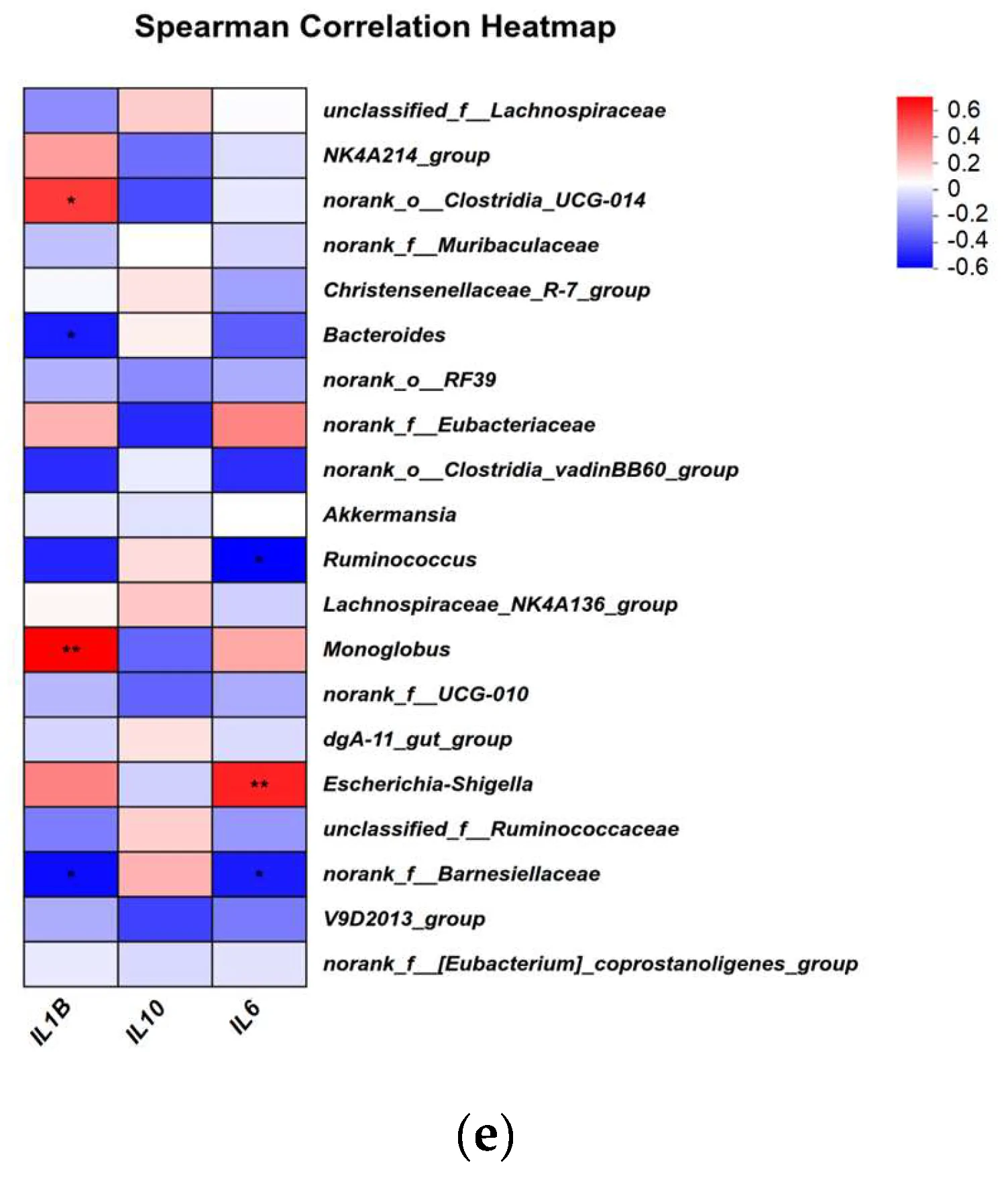
Pro treatment reverses gut dysbiosis and enriches beneficial commensals. (**a**) Relative abundance of bacterial taxa at the phylum level. (**b**) Relative abundance of bacterial taxa at the genus level. (**c**) Kruskal–Wallis analysis identifying key bacterial genera altered by LPS and restored by Pro. (**d**) LEfSe analysis showing characteristic bacteria in the three groups. (LDA score ≥ 3.5, *p* < 0.05). (**e**) Spearman correlation analysis of bacterial abundance and inflammatory cytokine levels. Red indicates a positive correlation, blue indicates a negative correlation, and color intensity reflects the correlation coefficient. *p* < 0.05 was considered statistically significant. * *p* < 0.05, ** *p* < 0.01, *** *p* < 0.001.

At the phylum level, analysis of taxonomic composition indicated that the gut microbial community was primarily composed of *Firmicutes*, *Bacteroidetes*, *Verrucomicrobia*, *Proteobacteria*, and *Desulfobacterota* (Figure 6a). At the genus level, significant alterations in certain bacterial groups were observed after Pro treatment (Figure 6b). In comparison to the LPS group, the addition of Pro exhibited significantly higher relative abundances of the *Lachnospiraceae_NK4A136_group*, *Bacteroides*, and *Ruminococcus* (*p* < 0.05). Concurrently, Pro treatment significantly decreased the relative abundance of *Escherichia–Shigella*, an opportunistic enteric pathogen (*p* < 0.05). To pinpoint the specific phylotypes driving these community shifts, Kruskal–Wallis and LEfSe analyses were conducted. The Pro intervention was associated with enrichment of potentially functional commensals, including *Ruminococcus*, *Christensenellaceae_R-7_group*, and *Lachnospiraceae_NK4B4_group* (*p* < 0.05, Figure 6c,d). *IL6* showed a negative correlation with the abundance of *Ruminococcus* and a positive correlation with the abundance of *Escherichia-Shigella* (*p* < 0.05, Figure 6e). Collectively, these results suggest that the LPS challenge led to considerable microbial dysbiosis, while Pro intervention effectively adjusted the abundance of crucial intestinal genera. These findings suggest a potential role for Pro in modulating the gut microbial community and restoring microbial homeostasis.

## 4. Discussion

Weaning stress serves as a key inducer of gastrointestinal dysfunction, mucosal barrier impairment, and growth retardation in commercial rabbit production. This study established a model of intestinal inflammation in weaned rabbits induced by LPS to systematically evaluate the protective and anti-inflammatory effects of Pro. The LPS challenge led to considerable damage to intestinal structure, inflammatory responses, and notable microbial dysbiosis, consistent with the typical pathological features of endotoxin-induced enteritis in young animals [16,28]. Importantly, the addition of Pro attenuated these pathophysiological abnormalities. These results suggest that the modulation of gut microbiota and enhancement of mucosal barrier functions may play a role in the intestinal protective effects of Pro. Interestingly, significant improvements were observed only with the 1% Pro dosage, while the 2% dosage did not yield better results. This could indicate a threshold or saturation effect, similar to what has been noted in nonlinear responses to amino acid dosages [29], where higher amounts do not provide further advantages and might even lead to metabolic or microbiota-related stress [30]. Future research should include dose–response studies with intermediate concentrations and mechanistic analyses to identify the ideal level of Pro supplementation. Additionally, while cage-level water intake was confirmed to be complete, individual water consumption was not monitored. This precludes the precise determination of individual Pro exposure and represents a limitation of the present study. Also, the lack of significant improvement in body weight gain may be partly attributed to the relatively short experimental duration, which may not have been sufficient to translate the intestinal protective effects into measurable growth benefits. Furthermore, the lack of a control group receiving only Pro (without LPS challenge) limits our capacity to differentiate between the direct effects of Pro on normal gastrointestinal homeostasis and its protective role in inflammatory models. It is also important to mention that this study exclusively involved male rabbits to reduce hormonal variability related to sex, leaving the applicability of these findings to females still uncertain. Sex-dependent differences in intestinal inflammation and barrier function have been suggested, with estrogen receptor β potentially contributing to colonic epithelial homeostasis [31]. Future studies incorporating both sexes may help clarify whether the protective effects of Pro are comparable between males and females.

Enhancing the function of mucosal barrier is a crucial phenotypic basis of Pro-associated intestinal protection and involves the restoration of physical, chemical, and immune barrier components. As a functionally essential amino acid, Pro serves as an indispensable substrate for collagen biosynthesis and is metabolized to glutamate and polyamines [32,33], which aid in the proliferation of intestinal epithelial cells and the repair of the mucosa. In this study, the addition of Pro markedly restored colonic epithelial architecture, reduced submucosal inflammatory cell infiltration, and normalized crypt architecture, thus reinforcing the structural integrity of the intestinal physical barrier. The increase in goblet cells following Pro treatment may also contribute to the mucus barrier by supporting mucus production and maintenance.

With respect to immune barrier regulation, Pro supplementation led to a notable decrease in the levels of the pro-inflammatory cytokines *IL1B* and *IL6* while upregulating the expression of the anti-inflammatory cytokine *IL10*. This reduction in inflammation corresponds with findings from studies on experimental colitis, indicating that both endogenous and exogenous regulatory molecules can reduce inflammatory damage to the intestinal lining by suppressing the release of pro-inflammatory cytokines [34]. Excessive inflammatory responses can promote oxidative degradation of tight junction proteins, thereby heightening paracellular permeability [35]. Consequently, restoring balance in inflammation may play a crucial role in maintaining the integrity of the intestinal barrier. However, since the current study primarily assessed cytokine expression at the mRNA level, additional investigations into the protein levels of cytokines and the activity of inflammatory signaling pathways are necessary to validate these conclusions.

The restoration of the intestinal epithelial barrier is closely interrelated with the maintenance of gut microbiota homeostasis [36]. Regulation of the microbiota–epithelial barrier plays a crucial role in sustaining the stability of the intestinal environment. The potential involvement of gut microbiota modulation in Pro’s protective effects is suggested by our data. Dysbiosis induced by LPS is commonly characterized by an increase in potentially harmful bacteria, such as those from the *Enterobacteriaceae* family, alongside a reduction in beneficial microbes that produce short-chain fatty acids (SCFAs) [37]. Our research indicated that Pro supplementation was associated with changes in the relative abundances of several bacterial genera, including *Ruminococcus*, *Christensenellaceae_R-7_group*, and *Lachnospiraceae_NK4B4_group*. The alterations in these key genera were also linked to enhanced mucosal barrier function and reduced inflammation, implying their potential role in sustaining microbial and intestinal homeostasis. Nonetheless, the correlation observed does not confirm that these specific taxa directly contribute to the protective effects of Pro.

The reported functional characteristics of these differentially enriched genera provide a plausible explanation for the observed protective effects in this study. As a major degrader of complex polysaccharides, *Ruminococcus* possesses a broad repertoire of carbohydrate-active enzymes (CAZymes) that hydrolyze indigestible dietary fibers and mucin-derived O-glycans into fermentable monosaccharides and oligosaccharides [38,39]. While this study did not directly assess bacterial enzyme activity or how substrates were utilized, the increased relative abundance of *Ruminococcus* following Pro supplementation may reflect a shift in the intestinal microbial environment that promotes carbohydrate fermentation. Such changes could potentially influence the availability of fermentation substrates and microbial metabolites, including SCFAs. However, this interpretation requires confirmation through metagenomic and metabolomic analyses. The *Lachnospiraceae_NK4B4_group* belongs to the *Lachnospiraceae* family, members of which have been reported to possess immunomodulatory properties and contribute to mucus layer homeostasis [40,41]. These traits align with the increased number of goblet cells and enhanced chemical barrier function noted in this research. Additionally, the *Christensenellaceae_R-7_group* has been linked to host metabolic health in earlier studies [42]. Its altered abundance during acute inflammatory stress may indicate shifts in host–microbiota dynamics, although the potential long-term metabolic implications of Pro supplementation remain undetermined based on the current findings.

Research involving pregnant sows aligns with our findings, indicating that the abundance of *Christensenellaceae_R-7_group* is positively correlated with the anti-inflammatory cytokine *IL10* while showing a negative relationship with the pro-inflammatory cytokine *IL6* [43]. This supports the association between this bacterial group and intestinal inflammatory status noted in our study. Additionally, we found a negative association between *Ruminococcus* and *IL6*, which is consistent with the findings of Wang et al. [44]. From a metabolic perspective, *Christensenellaceae_R-7_group* plays a significant role in acetate production [45], which can act as a precursor for butyrate-producing bacteria, such as those in the *Lachnospiraceae_NK4B4_group*. This metabolic relationship may collectively boost the generation of SCFAs in the gut. Therefore, the interactions among these bacterial groups could potentially affect the luminal SCFA profile. However, since SCFA concentrations were not assessed in our study, this proposed metabolic relationship remains speculative. Additional indirect support for the functional capabilities of this group comes from research on closely related strains within the same family. Notable strains like *Christensenella minuta DSM 33715* and *DSM 22607* have been reported to exert anti-inflammatory effects, maintain mucosal barrier integrity, and regulate metabolic balance in colitis models. These observations lend credence to the potential health benefits associated with members of the *Christensenellaceae* family [46,47]. Given these established insights, it is plausible to suggest that synergistic metabolic interactions could enhance the gut microecological environment and further aid in preserving mucosal barrier function.

## 5. Conclusions

Supplementation with 1% Pro through drinking water effectively alleviated LPS-induced intestinal injury in weaned rabbits. This protective effect was associated with improved growth performance, restored intestinal morphology, altered cytokine expression, and modulation of gut microbial community composition and structure.

## Figures and Tables

**Table 1:** Feed nutrient composition of basal diet.

Composition	(%)	Composition	(%)
Crude protein	16.83	Cystine	0.33
Crude ash	8.8	Arginine	0.88
Crude fiber	21.1	Lysine	0.99
Crude fat	2.8	Tyrosine	0.3
Water	8.1	Leucine	1.07
Neutral detergent fibers	40.1	Proline	0.88
Total phosphorus	0.62	Tryptophan	0.15
Calcium	1.0	Serine	0.75
Total amount of amino acids	14.14	Threonine	0.6
Phenylalanine	0.67	Aspartic acid	1.32
Alanine	0.71	Valine	0.7
Methionine	0.3	Isoleucine	0.54
Glycine	0.72	Histidine	0.39
Glutamate	2.81	Acid detergent fiber	25.6

**Table 2:** Scoring scheme for colonic inflammation mediated by luminal antigens.

Inflammatory Cell Infiltrate (%)		Epithelial Changes	Mucosal Architecture	Score
Severity	Extent			
Minimal	Mucosa	Minimal hyperplasia	-	1
Mild	Mucosa and submucosa	Mild hyperplasia, minimal goblet cell loss	-	2
Moderate	Mucosa and submucosa, sometimes transmural	Moderate hyperplasia, mild goblet cell loss	-	3
Marked	Mucosa and submucosa, often transmural	Marked hyperplasia with moderate to marked goblet cell loss	Ulcerations, crypt loss	4

**Table 3:** Quantitative PCR primer sequences.

Gene	Primer Sequences (5′–3′)	
	Forward	Reverse
*GAPDH*	AGGTCGGTGTGAACGGATTTG	GGGGTCGTTGATGGCAACA
*IL1B*	TGCCACCTTTTGACAGTGATG	ATGTGCTGCTGCGAGATTTG
*IL6*	TGATGGATGCTACCAAACTGGA	TGTGACTCCAGCTTATCTCTTGG
*IL10*	AGACCAAGGTGTCTACAAGGC	AGAGAGCTCTGTCTAGGTCCTG

## Data Availability

Data will be available on request. 16S rRNA sequencing data are available in the NCBI SRA database under the accession number PRJNA1508097.

## Supplementary Materials

The following supporting information can be downloaded at https://www.mdpi.com/article/10.3390/biology15171482/s1: Figure S1: The complete, uncropped original version of Figure 2b Western blot image. Figure S2: The immunofluorescence image of negative control. Table S1: Sequencing statistics and ASV counts of 16S rRNA gene amplicon sequencing for each sample. Table S2: Available composition of the raw materials.

## Abbreviations

The following abbreviations are used in this manuscript:

AB-PAS	Alcian blue–periodic acid–Schiff
ANOSIM	analysis of similarities
ASV	amplicon sequence variants
BW	body weight
GAPDH	glyceraldehyde-3-phosphate dehydrogenase
H&E	hematoxylin-eosin staining
*IL-6*	interleukin-6
*IL-10*	interleukin-10
*IL-1*β	interleukin-1β
LEfSe	linear discriminant analysis effect size
LPS	lipopolysaccharide
MAPK	Mitogen-Activated Protein Kinase
NF-κB	Nuclear Factor kappa-light-chain-enhancer of activated B cells
PAMP	pathogen-associated molecular pattern
PBS	phosphate-buffered saline
PCoA	principal coordinate analysis
POX	proline oxidase
Pro	L-proline
PVDF	polyvinylidene fluoride
qPCR	quantitative real-time polymerase chain reaction
SCFAs	short-chain fatty acid
TNF-α	tumor necrosis factor-α
TLR4	Toll-like receptor 4
ZO-1	zonula occludens-1
