# Supplementary material for: L-Proline Supplementation Alleviates Lipopolysaccharide-Induced Intestinal Inflammation in Weaned Rabbits by Modulating Mucosal Barrier Function and Gut Microbiota
Source: Biology (Basel). 2026 Sep 1;15(17):1482. doi: 10.3390/biology15171482 (PMC13565087; doi:10.3390/biology15171482)
Supplement: Supplementary file 1 [file biology-15-01482-s001.zip › biology-4494295-supplementary.pdf]

Supplementary Materials

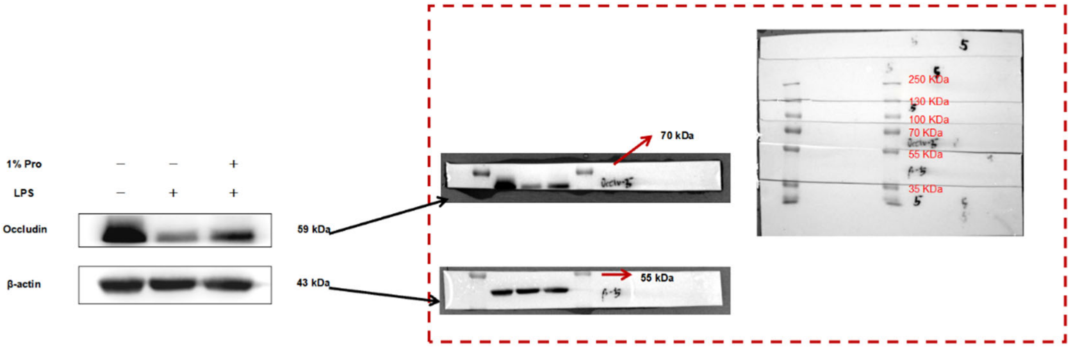

Figure S1. The complete, uncropped original version of Figure 2b Western blot image.

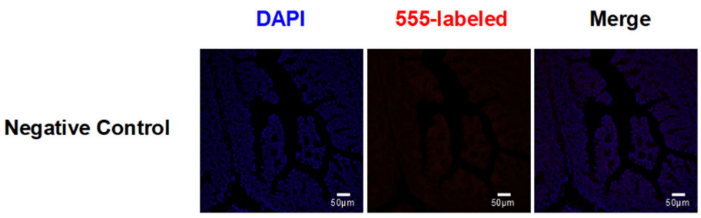

Figure S2. The immunofluorescence image of negative control.

Table S1. Sequencing statistics and ASV counts of 16S rRNA gene amplicon sequencing for each sample.

Sample ID	Effective Reads	ASVs	Rarefaction curves
CON1	92076	494	
CON2	80535	627	
CON3	100532	787	
CON4	86254	571	
CON5	88624	721	
CON6	98441	643	
LPS1	68616	409	
LPS2	114788	867	
LPS3	88730	685	
LPS4	89387	610	
LPS5	89962	638	
LPS6	88588	682	
LP1	94063	670	
LP2	96677	682	
LP3	106550	617	
LP4	83805	587	
LP5	94618	595	
LP6	96851	622	

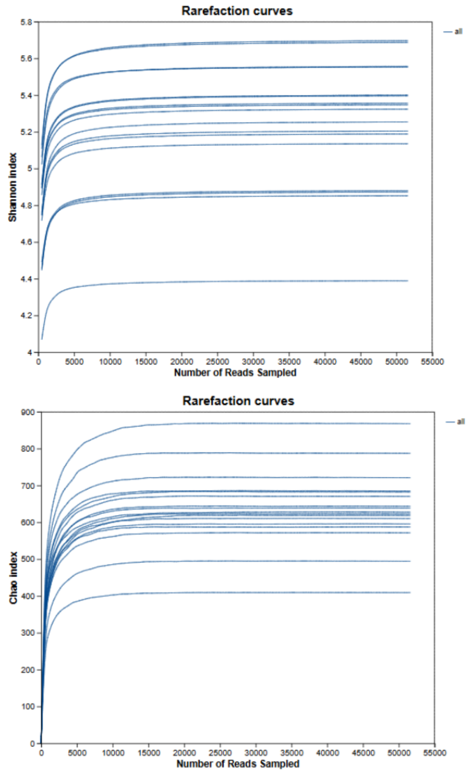

Notes: *Rarefaction curves were used to assess whether the sequencing depth was sufficient to characterize the microbial community in each sample. The curves approached a plateau at the tail, indicating that the sequencing data volume was adequate and reasonable.*

Table S2. Available composition of the raw materials.

Number	Ingredient composition
1	Maize
2	Alfalfa
3	Bean pulp
4	Calcium phosphate
5	Sodium chloride
6	Limestone
7	Vitamin premix
8	Trace mineral premix

Note: *The basal diet was a commercial pelleted feed. The exact percentage composition of each ingredient was not disclosed by the manufacturer. The nutrient composition is provided in Table 1 of the main manuscript.*
